# The obesity-breast cancer link: a multidisciplinary perspective

**DOI:** 10.1007/s10555-022-10043-5

**Published:** 2022-06-25

**Authors:** Emily N. Devericks, Meredith S. Carson, Lauren E. McCullough, Michael F. Coleman, Stephen D. Hursting

**Affiliations:** 1 Department of Nutrition, Gillings School of Global Public Health, University of North Carolina at Chapel Hill, Chapel Hill, NC USA; 2 Department of Epidemiology, Rollins School of Public Health, Emory University, Atlanta, GA USA; 3 Nutrition Research Institute, University of North Carolina at Chapel Hill, Kannapolis, NC USA; 4 Lineberger Comprehensive Cancer Center, University of North Carolina at Chapel Hill, Chapel Hill, NC USA

**Keywords:** Obesity, Breast cancer, Diet, Health disparities, Adipose, Immune

## Abstract

Obesity, exceptionally prevalent in the USA, promotes the incidence and progression of numerous cancer types including breast cancer. Complex, interacting metabolic and immune dysregulation marks the development of both breast cancer and obesity. Obesity promotes chronic low-grade inflammation, particularly in white adipose tissue, which drives immune dysfunction marked by increased pro-inflammatory cytokine production, alternative macrophage activation, and reduced T cell function. Breast tissue is predominantly composed of white adipose, and developing breast cancer readily and directly interacts with cells and signals from adipose remodeled by obesity. This review discusses the biological mechanisms through which obesity promotes breast cancer, the role of obesity in breast cancer health disparities, and dietary interventions to mitigate the adverse effects of obesity on breast cancer. We detail the intersection of obesity and breast cancer, with an emphasis on the shared and unique patterns of immune dysregulation in these disease processes. We have highlighted key areas of breast cancer biology exacerbated by obesity, including incidence, progression, and therapeutic response. We posit that interception of obesity-driven breast cancer will require interventions that limit protumor signaling from obese adipose tissue and that consider genetic, structural, and social determinants of the obesity–breast cancer link. Finally, we detail the evidence for various dietary interventions to offset obesity effects in clinical and preclinical studies of breast cancer. In light of the strong associations between obesity and breast cancer and the rising rates of obesity in many parts of the world, the development of effective, safe, well-tolerated, and equitable interventions to limit the burden of obesity on breast cancer are urgently needed.

## Epidemiology and classification of breast cancer

In 2020, breast cancer surpassed lung cancer as the leading cause of global cancer incidence in women [1]. Breast cancer is commonly stratified into molecular subtypes identified by immunohistochemistry for the presence of the estrogen receptor (ER), progesterone receptor (PR), and human epidermal growth factor receptor 2 (HER2/neu) [2]. Breast tumors with detectable ER, PR, or both, with or without HER2 amplification, are defined as luminal-like tumors [3]. Tumors with HER2 overexpression, but not ER or PR, are defined as HER2 + breast cancer [4]. Triple-negative breast cancer (TNBC) is defined by a lack of expression of all three receptors [5]. About 60–90% of all breast cancers express the androgen receptor, although its potential as a therapeutic target remains controversial [6–8].

Luminal A tumors, defined by high ER and PR expression without HER2 amplification, is the most common molecular subtype of breast cancer with the best prognosis. Luminal B, which expresses lower levels of ER and PR and can have HER2 amplification, often presents at a higher tumor grade and has a greater risk of recurrence [9]. Both luminal subtypes are generally responsive to endocrine-based therapies, making effective treatment options more widely available. However, intrinsic and/or acquired therapeutic resistance remains pervasive [10]. HER2 + positive breast cancer, accounting for about 20% of breast cancers, is often a more aggressive tumor subtype but generally responsive to HER2-targeted therapies [11].

While TNBC accounts for only 15–20% of breast cancers, this intrinsic subtype is particularly aggressive, more likely to metastasize, and due in part to the lack of targeted therapies, results in worse clinical outcomes including greater recurrence and lower rates of overall survival [12]. Breast cancer, particularly TNBC, is a heterogeneous disease, hence transcriptomic signatures have been developed to further stratify these cancers. Basal-like breast cancer (BLBC) is a particularly aggressive subtype, defined by gene expression profiles resembling that of basal and myoepithelial breast cells [13]. While not mutually exclusive, BLBC and TNBC are frequently coincident, with TNBC making up 50–75% of BLBC tumors, and about 80% of BLBC tumors lacking ER and HER2 expression [14]. Additionally, a subset of TNBC tumors are defined as claudin-low, characterized by a stem cell-like/undifferentiated phenotype, high expression of epithelial-to-mesenchymal (EMT) markers, low levels of genomic instability, and heightened infiltration of stromal and immune cells [15]. When categorized according to intrinsic subtype, 70% of claudin-low tumors are TNBC [16]. TNBC is also characterized by deficiencies in homologous recombination with the majority of *BRCA1* mutation-associated breast cancers classified as TNBC [17, 18].

## The obesity-breast cancer link: epidemiological evidence

In 2017–2018, the age-adjusted prevalence of obesity—defined as a body mass index (BMI) of 30 kg/m^2^ or greater—among US adults was 42.4% [19]. Obesity promotes incidence and progression of at least 15 cancer types, including breast cancer in postmenopausal women [20]. Adipose tissue becomes the predominant site of estrogen production after menopause. Hence, women with obesity have greater postmenopausal levels of estrogen and consequently greater exposure to estrogen’s protumorigenic effects [21]. Thus, obesity-mediated exacerbation of cancer is of pressing concern. Across all breast cancer subtypes, obesity is associated with worse disease-free survival and overall survival [22]. However, the relationship between obesity and breast cancer is complicated by subtype and menopausal status across the literature. Obesity in women who are postmenopausal increases overall relative risk of developing breast cancer to 1.33, largely driven by increased rates of ER + breast cancers [23]. However, being obese is also associated with postmenopausal TNBC incidence and progression [24, 25]. The relationship between obesity and HER2 + breast cancer is still incompletely understood. Obesity is consistently associated with worse overall survival in patients with early HER2 + breast cancer, but evidence on the link between obesity and advanced HER2 + breast cancer is heterogeneous [26–28]. Genetic predictors of obesity, including several single nucleotide polymorphisms associated with fasting glucose and insulin, also correlate with breast cancer risk independent of family history, age, or menopausal status, pointing to the importance of the relationship between obesity, breast cancer risk, and genetics [29, 30].

### Obesity and metastatic progression of breast cancer

Metastasis, the dissemination and growth of primary tumor cells in secondary sites, is the cause of 90% of tumor-related deaths in patients with breast cancer, with 5-year survival rates at 28% for affected patients [31, 32]. Typically, metastatic progression begins with local invasion of cancer cells from the primary tumor, first into the stroma surrounding the tumor and eventually to the neighboring normal tissue. Intravasation follows, as tumor cells expand their niche by entering lymphatic vessels to access the body’s systemic circulation [33]. As a distant site is reached, the cancer cells exit the bloodstream and proceed to adhere to the target organ. Metastatic outgrowth marks the final stage of metastatic progression, where quiescent tumor cells at distant sites are activated to begin proliferating [34, 35]. The mechanisms behind this activation are part of ongoing research.

For all subtypes of breast cancer, patients who are obese tend to have larger primary tumors at diagnosis and heightened risk of developing lymph node metastases [36]. Higher BMI predicts lower locoregional and distant recurrence-free survival among women with breast cancer [37], and increases association with overall mortality when compared to breast cancer patients at an ideal weight [38]. Indeed, patients with breast cancer and obesity are up to 46% more likely to have distant metastases 10 years after diagnosis [39]. Metabolic syndrome, defined in part by abdominal obesity, in patients with early breast cancer is linked to an increased risk of relapse as well as poor prognosis [40]. There are both biological and non-biological mechanisms contributing to the disparate outcomes for women with obesity and breast cancer, which have been expertly reviewed elsewhere [41].

Obesity expedites and exacerbates metastatic progression of breast cancer, supported by preclinical models [42–44] and clinical studies [39, 45]. Several leptin-mediated mechanisms behind this link have been established, including breast cancer invasion, migration, and immune regulation [46, 47], as well as cancer stem cell enrichment and mesenchymal stem cell dysregulation in the tumor microenvironment [31, 48]. Preclinical models of obesity demonstrate that increased myeloid-derived suppressor cell (MDSC) recruitment, collagen deposition, and changes in fibroblast phenotype in the lungs cooperate to create a favorable premetastatic niche for breast cancer [49].

## The obesity-breast cancer link: molecular mechanisms

### Obesity, adipose, and cancer interactions

**Fig. 1:**
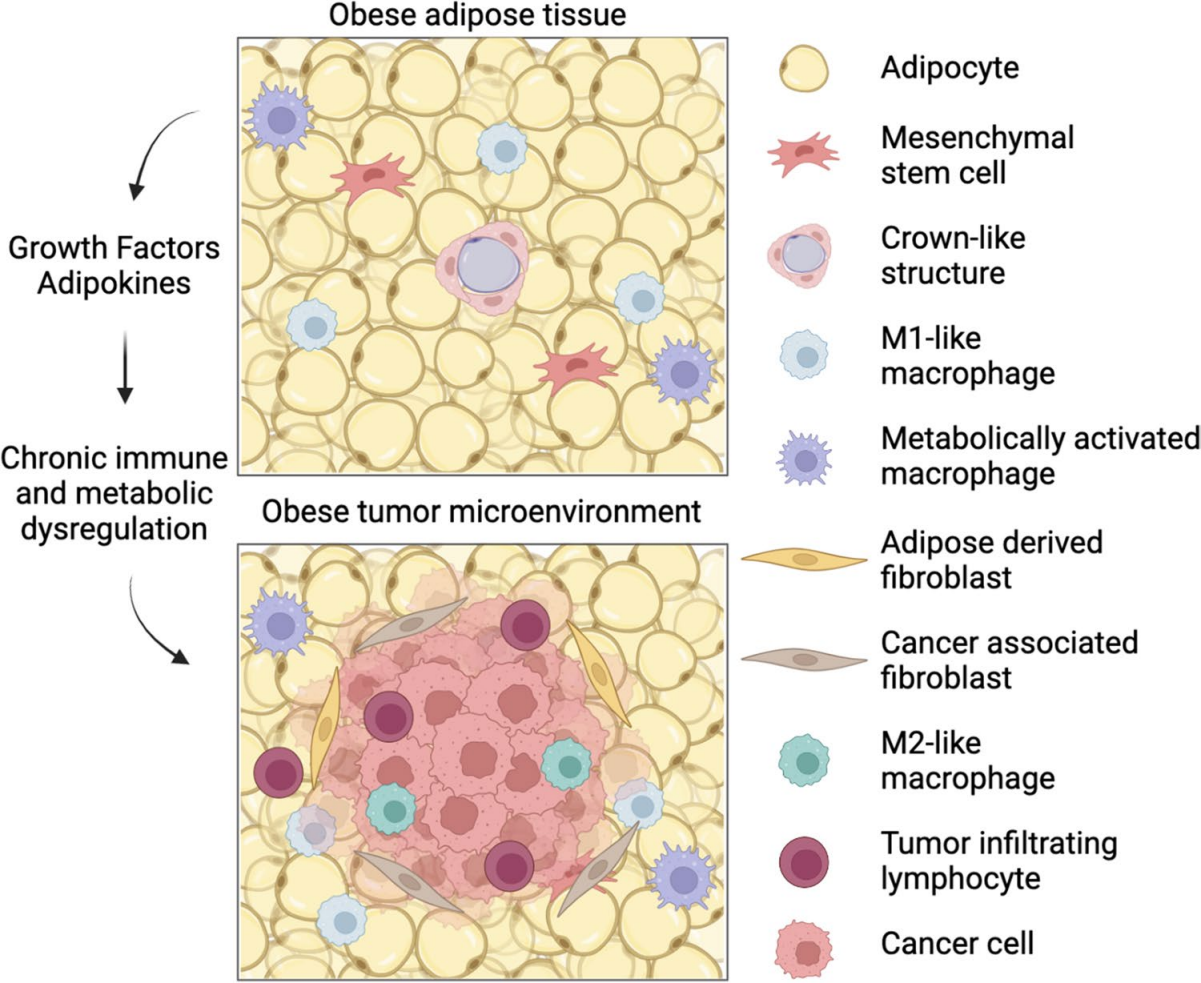
The impact of obesity on the tumor microenvironment. White adipose tissue from an obese host is composed of hypertrophied adipocytes, some of which become necrotic and induce formation of crown-like structures. Adipose tissue inflammation is furthered by M1-like and metabolically activated macrophages. Tumors developing adjacent to obese adipose tissue receive numerous inflammatory and metabolic signals from adipose and are marked by immunosuppressed tumor microenvironment with ineffective tumor-infiltrating lymphocytes and immunosuppressive M2-like macrophage polarization. Adipose tissue further contributes to the tumor microenvironment via recruitment and transdifferentiation of cancer-associated fibroblasts from mesenchymal stem cells, and adipocyte-derived fibroblasts from adipocytes

Separately, obesity and cancer are complex, integrating an incompletely understood combination of genetics, environment, and lifestyle. Hence, the relationship between obesity and cancer is immensely complicated. Despite the complexity, several mechanisms underlying the obesity-cancer link have been established. Increased white adipose tissue (WAT) mass is emerging as a nexus of tumor biology and metabolic and inflammatory dysregulation in obesity. WAT is composed of mature adipocytes, preadipocytes, endothelial cells, fibroblasts, pericytes, and immune cells [50]. Obesity also promotes hyperleptinemia, a result of dysregulated adipose tissue that can enhance inflammatory cytokine secretion [51]. In a murine model of renal cell carcinoma, hyperleptinemia has also been implicated in reduced efficacy of recombinant adenoviral/TLR agonist and anti-CTLA-4 checkpoint inhibitor immunotherapy [52]. WAT expands through adipocyte hypertrophy and hyperplasia in response to chronic nutrient excess, shifting the body’s energy balance signaling network and leading to elevated systemic insulin, estrogen, and adipokine signaling [53]. During adipose tissue expansion in the development of obesity, inflammation arises due to increases in immune infiltration, hypertrophic adipose tissue remodeling and angiogenesis, adipocyte necrosis, and dysregulated fatty acid flux due to heightened adipocyte lipolysis [54, 55]. Rapid adipocyte hypertrophy during adipose tissue expansion can create insufficient angiogenesis to achieve proper tissue vascularization, leading to hypoxic regions in WAT [56]. WAT hypoxia activates the transcription factor hypoxia-inducible transcription factor 1, which prevents preadipocyte differentiation and initiates adipose tissue fibrosis [57]. Adipose tissue macrophages engulf necrotic or damaged adipocytes to form distinct crown-like structures (CLS), a key feature of the pro-inflammatory process in adipose tissue [58]. Stressed adipose tissue, combined with hypoxia, promotes immune cell infiltration and stimulates inflammatory cytokine and chemokine release from resident macrophages in adipose tissue [59, 60] (Fig. 1). In addition to inflammatory signaling, adipocytes and their precursor mesenchymal stem cells (MSCs) support breast cancer progression by seeding the tumor microenvironment (TME) with critical supportive cell populations [61]. Adipose progenitor cells are more abundant in obese, relative to nonobese, mice [62], with greater levels recruited to the TME [63] supporting breast cancer growth and angiogenesis in vivo [64]. Cancer cells actively reprogram tumor-adjacent adipocyte matrix proteins and the inflammatory secretome, promoting the formation of cancer-associated adipocytes [65]. Cancer-associated adipocytes release free fatty acids into the TME [66], increase interstitial stiffness in breast adipose tissue [67], and enhance secretion of cytokines such as interleukin (IL)-6, interleukin (IL)-8, monocyte chemoattractant protein (MCP)-1, and tumor necrosis factor-alpha (TNFα), that promote inflammation [68]. In addition to supporting tumor cell migration, invasion [69, 70], and resistance to therapy [71, 72], cancer-associated adipocytes can transdifferentiate into adipocyte-derived fibroblasts in response to cues from the tumor. Adipocyte-derived fibroblasts, along with matrix metalloproteinases, modify extracellular matrix proteins to promote inflammation and tumor invasion [73–76]. Cancer-associated fibroblasts, which can arise from a variety of cell types including adipose-derived fibroblasts, are also key regulators of tumor development, metastatic progression, and therapy resistance [77].

In addition to modulation of adipocytes and other cells typically resident in adipose tissue, adipose-adjacent tumors actively recruit stromal cells, including MSCs, from elsewhere in the body, and reprogram their function through bidirectional communication with tumor cells to support metastatic progression [78]. Cancer cells induce lipolysis in adipocytes, releasing free fatty acids that are utilized by tumors for proliferation and migration [79] and stored within lipid droplets [66]. This transfer of fatty acids, stimulated by cytokines, sustains WAT inflammation [80] and occurs at a greater rate in breast cancer cells co-cultured with adipocytes from donors with obesity versus adipocytes from nonobese donors [79]. As breast tissue is composed of 90% WAT [81], and the human mammary epithelium is in permanent interaction with mammary adipose tissue [82], understanding the impact of excess WAT is imperative to resolving the relationship between obesity and breast cancer.

### WAT and adipokines

Elevated levels of endogenous sex hormones are associated with obesity and are correlated with a risk of breast cancer in postmenopausal women [83]. After menopause, estrogen production via activity of the key enzyme aromatase becomes noncyclical and occurs mainly in adipose tissue, exacerbating estrogen production in women with obesity [84]. Obesity not only elevates estrogen production in postmenopausal women, but also increases its bioavailability through reductions in sex-hormone binding globulin [85, 86]. Increased levels of pro-inflammatory cytokines, such as tumor necrosis factor-α (TNFα) and interleukin (IL)-6, further promote estrogen synthesis by inducing aromatase expression [87, 88].

WAT is a major endocrine organ, secreting hormones and growth factors, in addition to enzymes and metabolites. The WAT secretome is an important mediator of tumor exacerbation by obesity [81]. Adipokines, secreted by WAT, constitute a class of biologically active polypeptides with a broad range of endocrine, metabolic, and inflammatory functions [89]. Given the extensive interaction between adipocytes and tumor cells in the breast TME, adipokines play a critical role in the proliferative and invasive capacities of breast cancer [90]. While there are several adipokines (reviewed in [91]), two prominent examples are examined below.

Leptin, the polypeptide hormone primarily produced by adipocytes, is both synthesized and systemically circulates in proportion to adipose tissue mass [92]. Leptin levels are higher in patients with breast cancer compared to patients who are healthy, particularly in women who are overweight or obese [93]. Further, increased leptin associates with breast cancer risk at a standardized mean difference of 0.96 in a meta-analysis of 46 studies of over 13,500 women [94]. Leptin impacts breast cancer biology through a myriad of mechanisms that result in increased tumor volume and metastasis in preclinical and clinical models of breast cancer, including TNBC [33, 95]. Through activation of the PI3K/Akt pathway, leptin disrupts breast tissue epithelial polarity and promotes premalignant lesions [96]. High production of reactive oxygen species in TNBC is associated with lower antioxidant status to favor growth, survival, and inflammation in the presence of leptin [97]. In a patient-derived xenograft model of TNBC, leptin produced by obesity-altered adipose stem cells drove a prometastatic phenotype via upregulation of EMT-associated genes [31]. Increased leptin signaling in diet-induced obese mice results in tumoral cancer stem cell enrichment and mediates cell viability, migration, and invasion in triple-negative mammary tumor cells [48].

TNFα is a cytokine expressed in subcutaneous (and to a lower extent, visceral) adipose tissue [98] and preadipocytes [99]. In healthy breast tissue, TNFα contributes to cell proliferation and morphogenic branching [100]. As a key pro-inflammatory cytokine, TNFα is also expressed in monocytes and macrophages, and TNFα levels in adipose tissue rise 2.5-fold in individuals with obesity and have a strong positive correlation with hyperinsulinemia (*r* = 0.82) [101, 102]. TNFα promotes leptin secretion from adipocytes [103] and contributes to decreases in the anti-inflammatory adipokine adiponectin [104]. By inducing expression of aromatase and IL-6 in adipose tissue, TNFα also promotes estrogen synthesis [105]. TNFα promotes TNBC migration [106] and induces EMT in breast cancer stem cells while promoting a claudin-low phenotype [107] implicating this adipokine’s role in metastatic progression. Additionally, TNFα is linked to TNBC resistance to chemotherapy [108] and BLBC resistance to immune checkpoint inhibitors in vitro [109].

Thus, obesity dysregulates both endocrine and metabolic functions of WAT by promoting pro-inflammatory transformation, which is characterized by stromal remodeling, hypoxia, and altered immune profile. While significant progress has been made in understanding the relationships between these complex and intertwined processes, the relative contributions of each of these to the pathophysiology of obesity remains to be determined.

### Obesity and immune function

Protumor dysregulation of the prevalence and proportion of various immune cells in the obese TME and surrounding adipose tissue promotes angiogenesis, tumor growth, metastatic spread, and immune evasion culminating in adverse outcomes for patients with obesity and breast cancer [51, 110].

Adipose tissue remodeling that occurs with weight gain promotes recruitment of adipose tissue macrophages in both subcutaneous and visceral depots [111, 112]. Classically activated (M1-like) macrophages are more abundant in obese adipose tissue and form the characteristic CLS, named after their formation of a ring-like network of macrophages surrounding necrotic, hypertrophied, and dying adipocytes in breast adipose, and other WAT [58, 92, 112]. These macrophages disrupt adipocyte signaling, increase reactive oxygen species production, and promote secretion of pro-inflammatory cytokines [58, 113]. The infiltration of macrophages and the accompanying inflammation of breast adipose tissue of patients who are obese increases the risk of mammary carcinogenesis [114, 115].

In women who are obese, the breast adipose tissue produces CCL2 (also named MCP-1) and IL-1β to recruit macrophages and secrete CXCL12, resulting in increased CLS formation [116, 117]. The presence of CLS and inflammatory mediators in breast adipose tissue of women who are obese is associated with aberrant intracellular signaling and cellular dysfunction [84]. High densities of CLS are also independently associated with an increased risk of breast cancer, in addition to their negative impact on recurrence and survival [118–120].

Obesity-related drivers of immunological aging are characterized in part by premature thymic involution [121]. Lipid accumulation occurring with obesity can transform thymic fibroblasts into adipocytes, leading to reduced activity of the thymus [122]. This creates a reduction in the abundance, proliferation, and diversity of T cells, the essential players of cell-mediated immune response and adaptive defense against diseases like cancer [122, 123]. Gamma delta (γδ) T cells, defined by their γ and δ T cell receptors instead of the canonical α and β T cell receptors, have an increased pro-inflammatory population in adipose tissue from obese versus nonobese mice [124]. Obesity is also associated with higher pro-inflammatory CD4 + T_h_1 cells relative to static levels of anti-inflammatory T_h_2 cells and regulatory T cells (T_regs_) in adipose tissue [125]. However, due to aberrant insulin signaling, T_regs_ associated with obesity exacerbate adipose inflammation through alterations in cytokine production [126].

Obesity-related M1-like macrophages upregulate expression of programmed death-ligand 1 (PD-L1; an immune checkpoint protein) in TNBC, partially through enhanced secretion of IL-6 [127]. Classified by expression of the classical dendritic marker CD11c, these M1-polarized macrophages contribute to an immunosuppressive microenvironment by promoting T cell exhaustion [128]. While the breast TME comprises several types of immune cells, macrophages are the most abundant. Tumor-associated macrophages make up over 50% of TME macrophages [129] and are associated with aggressive features of TNBC tumors, including recurrence and metastases [130]. Leptin also activates IL-8 production in tumor-associated macrophages, driving tumor progression [131]. Metabolically activated macrophages are a pro-inflammatory population of macrophages unique to obesity and distinct from M1-like macrophages [132, 133]. Mammary adipose tissue macrophages from preclinical models of obesity produce inflammatory cytokines, induce a stem-like phenotype in breast cancer cells, and promote TNBC growth [132].

In adipose tissue, lower T_reg_ abundance, coupled with an increase in CD8 + T cells, creates an obesity-specific immune profile that promotes macrophage recruitment, inflammatory cytokine production, and consequently, can contribute to tumor progression [134, 135]. The increased leptin signaling characteristic of obesity increases PD-1 expression in T cells, resulting in T cell exhaustion and contributing to heightened inflammation [136, 137]. This immune dysfunction, however, correlates with greater response to PD-1/PD-L1 treatment in patients who are obese, including improvements in CD8 + /CD4 + ratios, metastatic burden and overall survival [136, 138–141]. This relationship is not universal, as reduced anti-PD-1 therapy efficacy occurs in patients with renal cell carcinoma who are obese [142]. The impact of obesity on checkpoint blockade inhibitor response remains unclear in TNBC.

Leptin receptors are highly expressed in activated T cells, impacting their sensitivity to nutrient availability [143]. Leptin plays an important role in the increased T cell dysfunction and PD-1 expression seen with obesity [136]. Indeed, leptin-STAT3 signaling in CD8 + effector T cell metabolism promotes fatty acid ß-oxidation while inhibiting glycolysis in a model of TNBC in high fat diet (HFD)-fed mice [144]. TNBC tumors from patients who are obese have higher expression of leptin, CXCR4, and CCR9 (receptors of CXCL12 and CCL25, respectively), which negatively correlate with CD8 + T cell infiltration, as compared to tumors from patients who are not obese [44]. FasL + granulocytic MDSC are increased with obesity via leptin signaling, causing CD8 + T cell apoptosis and resistance to immunotherapy [145].

Natural killer (NK) cells, type 1 innate lymphoid cells that have a large role in tumor response, fall into two distinct categorizations of CD56 expression. CD56^dim^ NK cells represent an activated phenotype and produce perforin and granzyme for cytotoxic functionality, while CD56^bright^ NK cells serve a more regulatory role [146]. NK cell populations are reduced both in number and activity in obesity, with a coincident decrease in the cytotoxic CD56^dim^ population and increase in the regulatory CD56^bright^ population [147, 148]. Like T lymphocytes, NK cells also express the leptin receptor [149]. Chronic exposure to the elevated leptin levels associated with obesity alter post-receptor signaling in these cells, lowering JAK2 phosphorylation and decreasing production of interferon-γ [149, 150].

MDSCs are an emerging mechanistic link between obesity and cancer [151]. Inflammatory signaling pathways promote the activation and downstream immunosuppressive function of MDSCs, driving their accumulation and activity in tumors as well as adipose tissue [151, 152]. Characterized by their expression of the cell surface markers Gr1 and Cd11b, MDSC populations accumulate threefold in adipose tissue of HFD-fed mice compared to their lean counterparts after 12 weeks on diet [153]. Increased levels of circulating leptin and exogenous lipids both drive immunosuppressive MDSC accumulation in adipose tissue and the TME, all of which work together to promote tumor growth [154–156]. Obesity, in part through crosstalk with leptin and availability of lipids in the TME, increases the presence of MDSCs and their PD-L1 expression to enhance tumor progression [154].

Advances in our understanding of the dynamic and complex relationships of the TME, including immune cells, has led to novel therapeutic strategies [157]. The chronically activated immune response characteristic of obesity can detrimentally impact therapeutic efficacy [136, 142]. However, as TNBC, relative to other breast cancer subtypes, generally has higher levels of tumor-infiltrating lymphocytes [158] and increased expression of immune checkpoint molecules, immunotherapy has become a promising avenue for PD-1/PD-L1 blockade treatments [159, 160]. An active area of research involves combining immunotherapies with other treatment modalities, including radiation, targeted therapies such as CDK4/6 inhibitors and PARP inhibitors, and cancer vaccines, to further improve their efficacy [161–164]. Another intriguing line of work in the immunotherapy field involves the interactions between obesity, immunotherapy response and several cancers [165].

Chronic inflammatory signaling in obesity limits immune responses to numerous diseases, including cancer. Rather than a uniform depression of function, obesity promotes altered function in a host of immune cells characterized by chronic systemic inflammation and limited antitumor immunity. Thus, ongoing work to delineate how obesity mediates disruption of antitumor immunity, and to identify interventions to mitigate this is of considerable importance.

## The obesity-breast cancer link: health disparities

### Obesity biases in breast cancer care

Due to the absence of targeted therapies, chemotherapy remains the standard of care for TNBC. As many as 40% of patients who are obese receive substantially lower doses relative to patients who are not obese due to dose calculation that does not incorporate body weight [166, 167]. Compared to patients who are not obese, patients with breast cancer who are also obese are more likely to have their doses capped at an arbitrary body surface area, even in the absence of toxicity expected at full intended doses [168, 169]. This phenomenon was shown to mediate the relationship between obesity and lower breast cancer-specific survival in a large observational cohort study [170]. However, even when dosing accounts for body weight, systemic chemotherapy is less effective in patients who are obese with breast cancer [41]. Tumor-associated adipocytes can induce multidrug resistance in breast cancer by upregulating a transport-associated protein that mediates doxorubicin efflux, a mechanism amplified by obesity [171]. In a murine model of TNBC, doxorubicin treatment is less effective in HFD-fed mice compared to control-fed mice due to changes in free fatty acid availability in mammary adipose tissue [172, 173].

Despite the improvements in our understanding of the complexity of factors contributing to obesity, coupled with its increasingly high prevalence, individuals with obesity continue to experience stigma and biased treatment in the healthcare setting [174, 175]. This is compounded by the lack of clinical intervention data in patients with cancer and obesity, with obesity status reported in only 5.3% of clinical trials of obesity-related cancers, including postmenopausal breast cancer [176].

### Obesity and breast cancer health disparities

Despite a relatively similar incidence of breast cancer among non-Hispanic White (NHW) and non-Hispanic Black (NHB) women, NHB women are ~ 40% more likely to die of breast cancer [177, 178] and have an overall 5-year breast cancer survival rate of 78.9% (compared to 88.6% among NHW women) [179]. While there have been substantial advances in breast cancer screening and treatment, resulting in an overall reduction in breast cancer mortality, declines are largely attributed to improvements among NHW women; the 2018-to-2019 change in the age-adjusted death rate was 19.2 *vs.* 18.8 per 100,000 among NHW women but unchanged among NHB women [180].

The etiology of racial disparities in breast cancer mortality are multifactorial and include contributing factors from social determinants of health and access to tumor biology and comorbidities. Obesity is an important consideration given the known association with risk, recurrence, and mortality across age categories and tumor characteristics [181]. Profound obesity-related disparities exist in the USA, where the prevalence rates of both obesity (49.6%) and severe obesity (BMI ≥ 40 kg/m^2^) (3.8%) among adults are most pronounced among NHB adults compared with other race and ethnic groups [19]. Notably, the obesity disparity is largely driven by NHB women; the 2017–2018 National Health and Nutrition Examination Survey showed that the prevalence of obesity among NHB women was 56.9% compared to 39.8% among NHW women [19]. Given the role of obesity in most aspects of cancer diagnosis, treatment, and progression [182], we posit that obesity could be a causal contributor to racial disparities in breast cancer outcomes, as further described below.

One factor in racial disparities in breast cancer mortality is differences in the presentation and prevalence of aggressive tumor subtypes. Specifically, adiposity increases the risk of postmenopausal ER + breast cancer and premenopausal ER-/TNBC [183]. It is well-established that NHB women compared to NHW women have a higher incidence of ER-/TNBC [184]. The Carolina Breast Cancer Study showed that BLBC occurs at a higher prevalence in premenopausal African American (AA) women (39%) compared to postmenopausal AA women (14%) and non-AA women (16%) [185]. BLBC, which progresses more quickly and has greater *TP53* mutations compared to the luminal A subtype, are more prevalent in NHB women and have an unfavorable prognosis. Indeed, AA women are more likely to carry a *TP53* mutation compared to White women [186].

While there is a higher prevalence of premenopausal TNBC in NHB compared to NHW women, luminal subtypes remain the most prevalent tumors among NHB women, accounting for approximately 75% of all breast cancer diagnoses [178]. After adjusting for age, NHB women with luminal A breast cancer have a 2.43 times higher rate of breast cancer mortality than their NHW counterparts [187]. Given that luminal A breast cancer — compared to TNBC — has better treatment options, insights are needed to understand drivers of such robust disparities in this relatively easier-to-treat subtype.

One potential mechanism of poor outcomes among NHB women with luminal A subtypes is obesity-related differences in DNA methylation that are associated with several clinical and histopathological features of breast cancer and clinical outcomes [188]. In a study that examined the association between obesity and DNA methylation in NHB and NHW women diagnosed with breast cancer, the authors detected interactions with ER status (*PSMB1, QSOX1,* and *PHF1*) and race (*TOMM20*) among the top 20 obesity-associated CpG cites*.* Additionally, differential methylation at the CpG sites of *TOMM20*, *PSMB1*, and *QSOX1* was associated with all-cause mortality, suggesting that obesity-related dysregulation may, in part, drive mortality differences [189]. Other obesity-related mechanisms may be relevant to differential tumor progression leading to racial disparities in outcomes, including local adipose tissue inflammation [190], diversity of the gut microbiome [191], and immune parameters, each of which can affect therapeutic effectiveness [192].

While there are several biological links between obesity and racial differences in breast cancer outcomes, biology alone cannot explain the persistent disparities across age and subtypes of breast cancer. Evidence suggests that obesity-related screening biases lead to delayed diagnoses, ultimately increasing cancer mortality in patients who are obese [182]. Acute and late treatment complications are more often seen among women who are obese, and—largely due to dosing uncertainty—there remains concern of treatment efficacy in women who are obese [193]. Notably, Black women, compared to their White counterparts, are more likely to be diagnosed at a later stage and less likely to receive stage-appropriate treatment [177]. The confluence of race and obesity-related biases may profoundly affect prognostic disparities. In addition to potential obesity-related race differences at the point of care, the inclusion of systemic inequities is essential to understand obesity-related drivers of breast cancer disparities. Collin and colleagues recently reported a 1.6-fold increase in breast cancer mortality among women residing in a redlined Atlanta neighborhood defined using present-day Housing Mortgage Disclosure Act data as odds of denial of a mortgage application for a residence inside the census tract compared with those outside of the census tract. While only 20% of NHW women diagnosed with breast cancer between 2010 and 2014 lived in a redlined census tract, 80% of NHB women diagnosed during the same time frame lived in redlined areas [194]. Redlining is an important driver of the built (*e.g.*, food deserts, green space, walkability) and lived (*e.g.*, environmental toxicants) environments, which profoundly affect adiposity. Additional research is desperately needed to explore the role of structural mechanisms in obesity-related breast cancer disparities.

## Dietary interventions to intercept obesity-mediated exacerbation of breast cancer

Obesity can lead to reduced efficacy of existing breast cancer therapeutics and increase treatment resistance [30, 195]. Patients with obesity experience greater risk of recurrence following several different endocrine therapies [196–199], and a patient’s BMI at diagnosis correlates with breast cancer recurrence across multiple subtypes [200]. Side effects during and after treatment, including lymphedema, also disproportionately impact women with obesity [201]. Patients with obesity also face greater risk of complications resulting from mastectomies, both with and without breast reconstruction surgery [202]. Cancer cell sensitivity to nutrient restriction, as well as their differing requirements for specific metabolites, constitute emergent hallmarks of cancer-targeted therapies [203]. Dietary interventions pose an inexpensive and effective way to manipulate availability of key nutrients for tumors and hence improve the effect of existing therapies, activate antitumor response mechanisms, and introduce tumor-specific toxicities [204]. Table 1 indicates some common dietary interventions used in clinical and preclinical studies.

**Table 1:** An overview of dietary interventions in human and mouse models

Diet	Diet composition (humans)	Diet composition (mice)	Reduction in caloric intake (humans)	Reduction in caloric intake (mice)	Periodicity
**Calorie restriction** [205, 206]	Typical diet of participant with micronutrients maintained	Vitamin-fortified isonitrogenous to ad libitum (AL) diet	20–25% reduction	20–40% reduction*	Chronic restriction without altered meal frequency in humans; Mice fed once daily
**Fasting** [207–209]	Variable	Standard diet	No food consumption for duration of fasting period, water allowed	No food consumption for duration of fasting period, water provided	Range from 12 h to weeks in humans; 24–60 h in mice
**Fasting-mimicking diet** [210–212]	Low protein (~ 10%), high fat (44–56%), high carbohydrate (34–47%): vegetable-based soups, broths, and tea	Standard diet for maintenance, proprietary FMD rodent diet with day 1 and days 2–4 caloric compositions	Day 1: ~ 1200 kcal; Days 2–4: ~ 200 kcal	Day 1: 7.67 kJ/g (~ 50% reduction); Days 2–4: 1.48 kJ/g (~ 90% reduction)	1–2 four-day cycles/month in humans; 10-day breaks (or complete body weight recovery) between four-day cycles in mice
**Intermittent energy restriction** [213, 214]	Mediterranean-style diet on days with no caloric reduction	Special diet with 2 × protein, vitamin, minerals, and fats for AL periods	~ 70–75% reduction for 2 days/week, AL for other 5 days/week OR 60–70% reduction every other day	Cycles of 50% CR for 3 weeks, followed by 3 weeks of no reduction	Chronic restricted cycles of either 5:2 or every other day in humans; cycles of 3 weeks AL, 3 weeks 50% CR in mice
**Time-restricted feeding** [215, 216]	Typical diet of participant; no intervention required to macronutrient composition	Highly variable	None required	None required	12-–20-h window of fasting every 24 h for humans and mice
**Ketogenic diet** [217, 218]	High fat (75–80%), very low carbohydrate (< 50 g/day, if possible), moderate protein (15–20%)	Customized rodent diets containing ~ 90% fat, 9–10% protein, and 0–1% carbohydrates	None required	None required	Chronic for humans and mice
**Mediterranean diet** [219, 220]	Vegetables (2–6 servings), fruit (1–3 servings), grains (< 8 servings), olive oil every meal (~ 37% fats, 33 g fiber/day)	Supplementation with omega-3 ethyl esters OR a Mediterranean-style formulated purified diet (highly variable)	None required	None required	Chronic for humans and mice

^*^Periodicity of calorie intake in CR in rodents is more impactful than the actual restriction itself [221]

### Chronic calorie restriction

Chronic caloric restriction (CR), typically defined as $\ge$ 10% reduction in caloric intake in humans and $\ge$ 20% reduction in rodents without malnutrition or the restriction of water, is an established mechanism for extension of lifespan and healthspan in clinical and preclinical models [205, 206, 222]. CR partially reverts some of the metabolic consequences of obesity in clinical trials [223, 224]. Postmenopausal women with an increased risk of breast cancer show improved hormonal (bioavailable estradiol, testosterone, and insulin) and adipocytokine (relative adiponectin/leptin, C-reactive protein) markers of breast cancer in serum and breast tissue with weight loss of more than 10% [225]. A meta-analysis of 59 preclinical studies concluded that calorie restriction is preventive of cancer development [226]. Indeed, CR reduces the incidence of several types of tumors in rodent and rhesus monkey models [226, 227]. CR prior to tumor induction slows primary tumor growth and reduces metastatic burden in a preclinical model of TNBC, and 30% CR imposed at the time of tumor induction in this same model synergized with radiation treatment to further suppress tumor growth [228–230]. A 30% CR dietary regimen also mitigates chemotherapy-induced inflammation and enhances radiation response by downregulating insulin-like growth factor (IGF)-1 receptor signaling, reducing metastatic tumor burden and improving overall survival in preclinical models of TNBC [207, 231]. These results were replicated with preclinical models of estrogen-responsive breast cancer, utilizing the fasting-mimicking diet (described below) and endocrine-based therapies [232]. Some of the metabolic protective effects of CR are mediated through periods of fasting, which arise when animals rapidly consume their daily calories and then fast for the remainder of each day [221]. However, this effect has not been examined in cancer models. Although CR is not nearly as well characterized in clinical studies as it is in preclinical models [233], human trials show promise in replicating the molecular and metabolic changes established in rodents [234]. Limited adherence to CR and unsafe weight loss, however, particularly in patients with advanced stage cancer, are of major concern for the success of this intervention [235, 236]. Larger clinical trials that incorporate dietary components, patient follow-up, and standardized treatment protocols are needed to more accurately assess the impact of CR on breast and other cancers [237].

### Time-restricted feeding

Time-restricted feeding (TRF), the practice of restricting time of calorie intake, rather than calories consumed, to an 8-–12-h window aligning with the circadian rhythm, is an emerging dietary pattern rapidly gaining scientific and cultural popularity [238]. As components of the circadian clock interact with nutrient-sensing pathways, inconsistent eating patterns and overeating can disrupt circadian regulation of endocrine and nutrient metabolism [239, 240]. By reducing evening energy intake and refraining from consuming food throughout the night, the TRF fasting regimen aligns meal times with the ideal postprandial hormonal response [241]. In a mouse model of postmenopausal obesity, TRF reduced body weight, improved glucose tolerance and insulin resistance, and reduced accumulation of fat in the liver [215]. These findings were corroborated by an 8-week clinical trial of time-restricted eating in participants who were overweight and obese, where a 10-h eating window resulted in clinically meaningful reductions in weight and improved fasting blood glucose [216].

There is growing evidence that circadian rhythm plays an important role in cancer pathogenesis, with chronic disruptions increasing breast cancer metastasis [242, 243]. Preclinical models of breast cancer demonstrate promising effects, with mice fed a high fat diet restricted to 12-h intervals having tumor burden reduced to that of control-fed mice [244].

### Fasting

In clinical research, fasting in humans is defined as significant-to-total reprieve of caloric intake for several hours to days [208]. Due to greater feasibility and improved adherence compared to CR, periodic fasting (PF) and short-term fasting (STF), maintained for periods of 48–72 h in rodents and 2–5 days in humans, may provide an effective compromise [235]. The synergistic effect of PF in combination with cancer treatment seen in preclinical studies [245] may be due to its effect on blood glucose levels, which are reduced more profoundly in preclinical models of PF relative to CR [246]. Engaging in STF induces a differential stress response between cancer cells and healthy cells, prioritizing cellular maintenance and repair in healthy cells, and exposing a vulnerability in cancer cells due to their inability to suppress growth-promoting pathways [246, 247]. The differential stress response provides a mechanism by which fasting can promote efficacy and tolerance of chemotherapy and radiation treatments [245, 246, 248, 249]. Additionally, fasting promotes stem cell self-renewal as well as regeneration of the blood, nervous system, muscle, and liver [235, 250]. In vitro data indicates TNBC sensitization to chemotherapy treatment with 24-h fasting [209]. Two clinical pilot studies with HER2-negative breast cancer patients demonstrate STF as a feasible, tolerated intervention in both trials and a reduction in the hematological toxicity of chemotherapy treatment in one trial [248, 251].

### Fasting-mimicking diet

The fasting-mimicking diet (FMD) is generally very low in proteins and carbohydrates, enriched in unsaturated fats and micronutrients, and maintained for a period of days in a cyclical fashion. It is an alternative regimen to a water-only fast with similar changes in stress resistance and blood glucose but greater tolerability and adherence [208, 250]. A combination regimen of FMD and endocrine-based therapies reduces IGF-1 receptor signaling, abates chemotherapy-induced inflammation, and enhances tumor response to radiation in preclinical models of estrogen-responsive breast cancer [232]. In vitro models of TNBC responded to a FMD, including reduced circulating glucose and IGF-1, with enhanced tumor immunogenicity and improved response to chemotherapy [210]. A clinical trial with 131 patients with HER2-negative breast cancer demonstrated that a FMD adhered to for 3 days prior to chemotherapy administration remained safe, effective, and lowered DNA damage in T cells as one metric of enhanced sensitivity to chemotherapeutic response [211]. Preclinical work has demonstrated the FMD to be effective against TNBC progression in combination with PI3K/AKT/mTOR inhibitors by potentiating kinase inhibitor response and reducing hyperglycemia, often a treatment-induced side effect [212]. Promising results from a clinical trial in which a majority of participants had breast cancer show that the FMD increases total and activated intratumor CD8 + T cells while decreasing circulating populations of immunosuppressive cells [252]. However, the impact of FMD on long-term patient survival, or its efficacy in patients with breast cancer and obesity, has not been demonstrated.

### Intermittent energy restriction

Intermittent energy restriction (IER) is a broadly-encompassing term that applies to recurring patterns of fasting, either rhythmic or arhythmic, in which calorie restriction is achieved by reduction in overall eating periods rather than meal sizes [253]. The periodicity of IER, as opposed to chronic CR, has greater adherence potential while maintaining comparable reductions in body weight in patients who are overweight or obese [213, 254, 255]. Reported benefits of IER include improved glucose regulation and stress resistance as well as reduced inflammation [256]. IER protects against in vivo tumor development in a spontaneous model of mammary carcinogenesis [257].

### Ketogenic diet

The ketogenic diet, composed of high fat, moderate protein, and very low carbohydrates, acquired its name in pursuit of ketogenesis: utilizing fatty acids, metabolized as the ketone bodies β-hydroxybutyrate and acetone, rather than glucose for energy [217]. The mechanistic basis for a ketogenic diet reducing breast cancer incidence and overall disease burden lies within decreased insulin signaling and overall reduced inflammation seen with prolonged nutritional ketosis [258]. Although some data exists demonstrating the feasibility of a ketogenic diet intervention during radiotherapy treatment for patients with breast cancer, such as boasting reductions in body weight and fat mass [259], there is little evidence that a ketogenic diet reduces tumor burden in patients with breast cancer. After 12 weeks of the ketogenic diet, quality of life and physical activity scores were not improved in a study of 80 patients with breast cancer undergoing chemotherapy [260]. However, in vitro and in vivo supplementation with the ketone body β-hydroxybutyrate increases tumor growth in breast cancer models, presumably by providing fuel for oxidative mitochondrial metabolism [261, 262]. However, β-hydroxybutyrate has no effect on TNBC proliferation or response to treatment via chemotherapy or radiation in vitro [263].

### Mediterranean diet

Growing evidence suggests that the consumption of a Mediterranean dietary pattern has a protective effect against many chronic diseases and cancers including breast cancer [264]. The key characteristics of a Mediterranean diet include high consumption of fruits and vegetables such as green leafy vegetables, legumes, nuts, and cereals; moderate intake of fish and other meats; and low intake of sweets and eggs [219]. The majority of studies examining the protective effect of Mediterranean diet on cancer progression are largely observational studies followed by randomized clinical trials [265, 266]. Overall, high adherence to a Mediterranean dietary pattern is associated with decreased risk of cancer mortality [267] and risk of developing breast cancer [268]. While epidemiological studies suggest an inverse relationship between adherence to a Mediterranean dietary pattern and breast cancer risk, the evidence regarding breast cancer subtype is limited [265, 269]. The results of one systematic review suggest an inverse association between a Mediterranean diet pattern and breast cancer risk in postmenopausal women, and particularly postmenopausal TNBC; however, results are mixed [265]. Olive oil, the main dietary fat in Mediterranean diet, has anticancer effects in experimental studies. For example, a high extra virgin olive oil diet in a rodent model of mammary carcinogenesis increases tumor latency and decreases tumor volume, multiplicity, and incidence [270]. Additionally, olive oil consumption is associated with a lower odds of developing breast cancer [271]. Furthermore, polyunsaturated fatty acids present in a Mediterranean dietary pattern, particularly omega-3 fatty acids and specifically docosahexaenoic acid (DHA) and eicosapentaenoic (EPA), have antiproliferative effects in preclinical models of TNBC [272]. Indeed, supplementation with EPA + DHA ethyl esters reduces mammary tumor growth in obese postmenopausal (ovariectomized) mice [220].

### Dietary considerations

Reducing calorie intake, through fasting or calorie restriction, prior to chemotherapy treatment reduces side effects commonly associated with chemotherapy [273] and may thereby improve quality of life for patients [251, 274, 275]. However, due to heightened risk of adverse effects associated with weight loss and alterations to inflammatory response that can occur with calorie restriction, widespread clinical intervention remains challenging [247]. Additionally, adherence to chronic CR has proven challenging for humans, with sustainability dropping off after ~ 20 weeks for participants who are not obese [276]. A long-term dietary intervention study of participants who were overweight or obese showed adherence drops of 38% for alternate-day fasting and 29% for CR over 1 year [254]. As food intake is neurologically regulated, several mechanisms are in place to drive food consumption during periods of deprivation, causing psychological stress and negatively impacting mood if restriction is too severe or extends for too long [253, 277]. Physiological changes concurrent with obesity, such as increases in orexigenic acetyl-CoA binding protein and disruption of leptin- and ghrelin-mediated appetite signaling cues, provide further barriers for adherence to restrictive diets [278, 279].

One solution to achieve the benefits of dietary restriction without encountering barriers to adherence or disturbing energy balance are calorie restriction mimetics (CRMs). The main objective of CRMs is to induce autophagy, which protects against cellular stress and damage, mobilizes energy reserves, and removes intracellular waste or debris [280]. By optimizing energy and redox metabolism, activating this endogenous mechanism may help cells avoid malignant transformation [281] or improve antitumor immunity through autophagy induction [282].

## Conclusion

Obesity promotion of the incidence and progression of numerous cancers, including breast cancer, poses a significant public health hazard. Delineation of the mechanisms through which obesity drives cancer progression or immune evasion is critical for the development of interventions to effectively disrupt obesity-driven cancer with minimum toxicity. Dietary interventions remain of considerable interest due to their co-targeting of metabolic disruptions. Breaking the obesity-breast cancer link will require interventions that limit the protumor effects of obesity-associated adipose dysregulation and that consider other biological and genetic mediators and structural determinants of health disparities. Moreover, given high and rising rates of obesity in many parts of the world, emphasis should be placed on the development of safe and effective interventions that are acceptable and accessible to all women to reduce the burden of obesity on breast cancer.
